# Prevalence, factors associated and knowledge of probable dementia among adults 50 years and over attending a faith-based geriatric center in Uganda

**DOI:** 10.1038/s41598-023-33948-9

**Published:** 2023-04-25

**Authors:** Machuor Daniel Arok Awuol, Besigye K. Innocent, Ayenyo Winfred

**Affiliations:** 1 Department of Family Medicine, Makerere University College of Health Sciences, Kampala, Uganda; 2 Soroti Regional Referral Hospital, Sororti District, Uganda

**Keywords:** Diseases, Health care, Medical research, Neurology, Risk factors, Signs and symptoms

## Abstract

Dementia is on the rise due to increasing proportion of old people in sub-Saharan Africa (SSA). Although dementia is misattributed to normal ageing or supernatural causes in SSA, it is a brain disease with well-established etiologies. Limited knowledge and understanding of dementia means that many older people are suffering without seeking help and are undiagnosed and untreated. The aim of this study was to determine the prevalence and factors associated with probable dementia and to describe the knowledge of the disease among adults 50 years and over attending a faith-based geriatric center in Uganda. This was a cross-sectional study using quantitative methods. A total of 267 adults 50 years and over attending a faith-based geriatric center in Mukono, Uganda were interviewed between 1 April and 15 May 2022. Interviews were administered using the Early Dementia Questionnaire (EDQ) and Dementia Knowledge Assessment Scale (DKAS). Data on participants’ socio-demographics, economic income, living arrangement, history of smoking, alcohol consumption, exercise and past medical history was collected using an additional questionnaire. Adults 50 years and over were included in the study. Logistic regression analyses were done. Probable dementia was 46.2% in the sample. The most common symptoms of probable dementia in the order of their severity were memory symptoms, (β co-efficient β 0.08, *p* < .001), physical symptoms (β 0.08, *p* < .001), sleep disturbances (β 0.81, *p* < .001) and emotions (β 0.04, *p* < .027). The final degree of association as determined by adjusted PR in the multivariable model revealed that only older age (aPR = 1.88, *p* < .001) and occasional/non-believer (aPR = 1.61, *p* = .001) remained significantly related to probable dementia. The study also found that 8.0% of the participants had optimal knowledge of dementia. There is high burden of probable dementia among adults 50 years and over attending a faith-based geriatric center in Mukono, Uganda. Factors associated with probable dementia are older age and being an occasional/non-believer. Knowledge of dementia among older adults is low. There is need to promote integrated early dementia screening, care and educational program in primary care to reduce the disease burden. Spiritual support would be a rewarding investment in the lives of the ageing population.

## Introduction

Dementia is a global public health concern of greater priority. It is a syndrome in which there is a deterioration in memory, thinking, behavior and ability to perform everyday activities^1^. Dementia is caused by variety of diseases that primarily or secondarily affect the brain, such as Alzheimer’s disease, vascular dementia, fronto-temporal lobar degeneration and dementia with Lewy bodies^2^. Without prior awareness, the symptoms of dementia may not be recognized by the person living with dementia or the family, and some of the early manifestations such as memory loss, functional disability or emotional liability may be thought of as normal ageing. However, old age is a strong predictor of dementia^3^ and those with other modifiable risk factors such as physical inactivity, alcohol consumption, smoking, unhealthy diets, diabetes mellitus, hypertension, HIV/AIDs, and heart disease are at increased risk of developing the disease early^4^.

There are 50 million people with dementia worldwide, with nearly 60% living in low- and middle-income countries. This number is estimated to reach 152 million by 2050^1^. A report by Alzheimer’s disease International estimated the age-adjusted dementia prevalence in sub-Saharan Africa to be 7.2% among those aged 60 years and over^5^. Uganda is a developing country with a population of about forty-five million people of whom approximately 4.6% is over 60 years^6^. This population is projected at 5.4 million by 2050. A population-based study in Southwestern Uganda reported that 20% of the sample screened positive for dementia^7^.

The rising prevalence of dementia is due to growing population of older persons in sub-Saharan Africa where the disease is misattributed to normal ageing and supernatural causes^4,8^. Lack of insight is a feature of dementia and people may be unaware that they have a problem and will resist seeking help. Diagnosis for dementia is therefore, made when a person or someone close first identifies the problem, associates it with dementia and makes a decision to seek medical help^9^. This decision is influenced by knowledge of dementia symptoms and risk factors among others and may take several months, with 91% of global cases being diagnosed late^3^.

Increasing awareness and knowledge of dementia is critical in reducing its risk factors among the at-risk population. Good knowledge of dementia symptoms and its risk factors is shown to increase early seeking of help for dementia diagnosis and treatment which ultimately results to improved patient’s quality of life and vice-versa. Knowledge of dementia refers to knowing what dementia is, its symptoms, risk factors and what is done when one has it. A study on the knowledge of dementia in South Africa showed only 10% reported knowing what dementia as^10^. Assessment of knowledge of dementia among undergraduate university students in Uganda reported a significantly poor knowledge of the disease^11^. In addition, there is a paucity of information on the knowledge of dementia in the general population.

This study therefore, set out to determine the prevalence and factors associated with probable dementia and to describe the knowledge of the disease among older adults attending a faith-based geriatric center in Mukono, Uganda. Detecting dementia early will allow patient and the family to seek help for treatment initiation, which is important in delaying disease progression, improving patient’s quality of life and reducing caregiver’s burden.

## Methods

### Study design

This was a cross-sectional study using quantitative methods.

### Study setting

The study was done in Reach One Touch One Ministries (ROTOM) Health Center-Mukono, Central Uganda. It is a private, faith-based geriatric center. ROTOM was established in 2003 as a non-denominational Christian ministry dedicated to meeting the spiritual, social, physical and psychological needs of the elderly and their dependents so they may live dignified, independent and hopeful lives and has grown today to become the leading organization meeting the needs of the elderly people in Uganda. The center is located 15 km north-east of Mukono town. Mukono is a District, with a total area of 2,986.47 Square kilometers, lying in the Central region of Uganda, located 21Kms East of Kampala City and sharing borders with the Buikwe District in the East, Kayunga along River Sezibwa in the North, Luwero in the North West, Kampala and Wakiso in South West, Tanzania—Lake Victoria in the South with the Islands of Buvuma District.

Approximately 1000 older persons do access health services in the center. Adults 50 years and above are eligible to attend the services in the center. About 915 of the older persons currently have sponsorship, managed through the center, and do not have to pay for services. This group resides in the rural areas of Mukono District. The second category of older adults are those without sponsorship, who come from surrounding districts of Kayunga, Buikwe, Wakiso and Luweero to seek health services at the center. While Mukono is a multiethnic town, majority of people who attend center speak Luganda. The center is open for 24 h for all the days of the week, and it averagely receives 15 older clients per day who attend for various needs. These services include general outpatient and inpatient care, medical outreach and screening, home visits, home safety, hygiene and sanitation drive, fellowships for seniors, food and income enhancement and chronic care services. Specific days are designated for special activities such as fellowships, distribution of food and non-food items, home visitations and sanitation drives. Emergencies are not seen at the center and patients self-refer to the medical service. ROTOM is therefore, not a standard PHC service as it serves the older adult population. It is however, important to note that, while older persons can access these various services in the center, there are limited screening and care services for dementia. The staff that run the health facility include a manager/medical officer in-charge, clinical officer, monitoring and evaluation officer, four community nurses, a facility-based nurse, laboratory technician, pharmacist assistant and eight formal caregivers who support the older persons who have no caregivers during daycare activities.

### Study population

The target population were adults 50 years and over attending clinics for the elderly. The accessible population were adults 50 years and over attending ROTOM Health Center-Mukono. The study unit were adults 50 years and over and their accompanying caregivers attending the center and fulfill the eligibility criteria. The inclusion criteria were adults 50 years and over and their accompanying caregivers attending the center during the study period. The exclusion criteria included participants who screened positive for pseudodementia.

### Sample size

A sample size of 271 was used in the study. This was determined using Kish Leslie formula (1960) for estimating prevalence in cross-sectional studies. The parameters used in sample size estimation were: 95% level of confidence, 5% level of precision and proportion of dementia of 20%^7^.

### Sampling procedure

Consecutive sampling was used. Every adult 50 years and older and their accompanying caregivers that presented to ROTOM health center-Mukono were included in the study. The center averagely receives 15 older adults in a day. The first 10 patients on the daily attendance register were recruited to participate in the study. The second patient on the register for that day was sampled as the first study participant. This method of sampling was used because there is no designated clinic for dementia care with the patients being walk-in patients, at different times of the day.

### Data collection instruments

All participants were screened for pseudodementia due to depression, using the Geriatrics Depression Scale (GDS) before they were subjected to dementia assessment. The GDS score of 5 or more was used to consider presence of pseudodementia. About 4 participants screened positive for pseudodementia and were excluded from the study because pseudodementia tends to impair cognition and this would result to false positives.

Data was collected using a combination of questionnaires: The Early Dementia Questionnaire (EDQ) and Dementia Knowledge Assessment Scale (DKAS).

The prevalence of probable dementia was determined using the EDQ. This tool has been used and validated in primary care settings of Malaysia. EDQ is not affected by patient’s cultural and educational background and therefore does not need cross-cultural validation and adaptation. In addition, EDQ measures early dementia more effectively than Mini Mental State Examination (MMSE) which is rather very good in measuring severe dementia. This makes EDQ the best screening tool for dementia in primary care settings. However, MMSE is still the gold standard for diagnosis of dementia. It uses the symptoms of dementia, with 20 questions. These include: memory symptoms**,** concentration, physical symptoms**,** emotions**,** sleep disturbances and symptoms such as confusion and awareness of outsiders about changing behaviour. Scoring of the EDQ was done through a Likert Scale response ranging from 0–3. The score 0 describes never, 1 seldom, 2 sometimes and 3 always. The minimum score was 0 and the maximum 60.

To determine severity of symptoms, the scores were based on the symptoms a patient had in a week for the past 2 years. A score of 0–7 indicates a patient was normal and a score of 8 or more shows the patient had probable dementia. This cut-off point of 8 was based on the fact that eight of 20 questions of dementia symptoms are identified as early symptoms of probable dementia.

Knowledge of dementia was determined using the DKAS which provides a valid and reliable measure of knowledge characteristics of diverse populations^12^.

The DKAS comprises statements about the syndrome that are factually correct or incorrect, which were developed on the basis of a literature review and international Delphi study with dementia experts. Respondents answer on a modified Likert scale with five response options: false, probably false, probably true, true, don’t know. Preliminary study identified four hypothesized components/subscales within the measure that have been defined as Causes and Characteristics (dementia pathology and terminal course), Communication and Behaviour (how a person with dementia engages with the world), Care Considerations (dementia symptoms relevant to the provision of care), and Risks and Health Promotion (risk factors and conditions that are associated with or mistaken for dementia)^13^.

However, in this study, 5 subscales/indicators of dementia knowledge were used: The knowledge of risks factors, onset, progression/symptoms, and screening and treatment pathways. To determine the knowledge of dementia, a statement about syndromes that are factually correct was used on a set of 27 questions, with a response of either true or false.

Data on participants’ socio-demographics, economic income, living arrangement, history of smoking, alcohol consumption, exercise and past medical history of hypertension, diabetes mellitus and HIV/AIDS was collected using an additional questionnaire during the interviews.

### Data collection procedure

Data was collected from 01/04/2022 to 15/05/2022. After informed consents were obtained from study participants and their accompanying adults, the study questionnaires were administered by trained research assistants who are employees of ROTOM health center and hold diploma in nursing. A total of 267 questionnaires were answered by a dyad of older adults and their caregivers. The caregiver was either a spouse, a child (above 18 years), or other close relative. A total of 267 caregivers that accompany the participants were interviewed. This was to ensure completion of questionnaires, especially by caregivers of older adults who might be experiencing memory loss. Interviews took a maximum of 30 min. Each filled questionnaire was cross-checked for completeness before the interview was terminated and clarifications sought. All methods were carried out in accordance with relevant guidelines and regulations.

### Data analysis

Data was entered on EpiData version 3.1, exported and stored on Microsoft Excel 2010. A dataset from 264 completed questionnaires was presented in excel format for analysis. 3 observations with missing data were dropped. Data was exported to SPSS version 12.0.

Descriptive statistics were computed and summarized using frequencies (for categorical variables) and medians (for numerical variables) with their respective inter-quartile ranges. Prevalence was calculated to assess the magnitude of probable dementia.

Bivariate and multivariable analysis using logistic regression were done to determine factors associated with probable dementia. Variables with *p*-values < 0.2 were considered for multivariable analysis. A step by step process was followed. The features were selected and normalized. A loss function was selected and hypothesis formulated. The cost and loss function were minimized using the gradient descent algorithm and then the hypothesis was tested. The variables in the final multivariable model were significant when *p* < 0.05. The measure of association was reported as prevalence ratio (PR) with corresponding 95% CI p-value. We used PR instead of odds ratio because the prevalence of the outcome is common (> 10%). Using odds ratios would under estimate the measure of effect (prevalence ratio) in the sample.

The knowledge of dementia was described using descriptive statistics. All the correct responses were coded = 1 and false responses were coded = 0. It was expected that if a person passed all the responses, the total score would be 27. All the scores per study IDs were summed up to get the total score for each subject. The median score of 19 was then identified. Participants below median score were categorized as having less than optimal knowledge and those above were categorized as having optimal knowledge.

Participants’ overall knowledge was categorized using modified Bloom’s cut-off point, as good if the score was between 80 and 100% (19–27 points), moderate if the score was between 50 and 79% (10–18 points), and poor if the score was less than 50% (< 10 points).

### Ethical approval and consent to participate

Permission to do the study was sought from the Department of Family Medicine at Makerere University. Approval was obtained (Number: Mak-SOMREC-2021-224) from School of Medicine Research and Ethics Committee (SOMREC), College of Health Sciences, Makerere University. Administrative support was sought from Mukono District Health Office, Mukono and Monitoring and Evaluation Office, ROTOM Health Center-Mukono. Written informed consents were obtained from each study participants. In case of illiteracy or lack of capacity, a consent was obtained from the accompanying caregiver. If both the participant and caregiver were illiterate, the consent was obtained verbally from the participant after a brief explanation of study procedure by the trained research assistants. Confidentiality was ensured. Names of study participants were not written on the questionnaires—only research numbers were used. The completed questionnaires were kept under lock and key and only accessed by the research team. The COVID-19 preventive measures, including use of masks, hand washing and social distancing were fully observed during the entire study period. Participants who screened positive for dementia were referred to Mukono General Hospital (MGH) for care. However, we do not expect much support from MGH as there are no dedicated dementia care services offered by the facility.

## Results

### Characteristics of study sample

A sample of 267 participants was tested for probable dementia, giving a response rate of 98.5%.

The mean age of participants was 72.2 years (SD 10.8). Majority were female, 71% (*n* = 186); living in rural areas, 83% (*n* = 219); formally unemployed, 91% (*n* = 239); earning income less than 500,000 Ugandan Shillings (230 United States Dollars), 96% (*n* = 252); living with someone, 91% (*n* = 241) and non-smokers, 88% (*n* = 231). More than half were practicing believers, 60% (*n* = 157) and exercising occasionally, 52% (*n* = 132). Half were widowed, *50%* (n = 132) and had attained primary level of education, 50% (n = 131). Hypertension, 38% (*n* = 101) and alcoholism, 17% (*n* = 45) were commonly reported unlike diabetes mellitus, 0.8% (*n* = 2); heart disease, 0.4% (n = 1) and stroke, 0.8% (*n* = 2). Table 1 summarizes the sociodemographic and patient characteristics.

**Table 1:** Socio-demographic and patient characteristics of 264 participants recruited in the study.

Variable	Frequency(N = 264)	Percent (%)
Social demographic characteristics		
Age		
50–70 years	117	44.3
71–85 years	124	47.0
> 85 years	23	8.7
Sex		
Female	186	70.5
Male	78	29.5
Residence		
Rural	219	83.0
Urban	45	17.0
Marital status		
Single	1	0.4
Divorced	12	4.6
Widowed	132	50.0
Married	101	38.3
Separated	18	6.7
Level of education		
No formal education	72	27.3
Primary education	131	49.6
Secondary education	47	17.8
A level/college/university	14	5.3
Formal employment		
Employed	15	5.7
Unemployed	239	90.5
Retired	10	3.8
Income level		
Less than 500,000 shillings	252	95.5
Above 500,000 shillings	12	4.5
Practicing faith		
Practicing believer	157	59.5
Occasional believer	104	39.4
Non-believer	3	1.1
Current living arrangement		
With family/friends/others	241	91.3
Alone	23	8.7
Smoking		
Smoker	5	1.9
Non-smoker	231	87.5
Ex-smoker	28	10.6
Exercise		
Daily	78	29.6
Occassionally	136	51.5
Not at all	50	18.9
Past medical history		
Hypertension		
No	163	61.7
Yes	101	38.3
Diabetes mellitus		
No	262	99.2
Yes	2	0.8
Heart disease		
No	263	99.6
Yes	1	0.4
Stroke		
No	262	99.2
Yes	2	0.8
Alcoholism		
No	219	83.0
Yes	45	17.0

SD is standard deviation.

### Prevalence of probable dementia

Probable dementia was 46% in the sample (PR, 95% CI 0.46; 0.40–0.52). Among those who screened positive for probable dementia, 84.4% were adults 50–85 years old. Those aged 85 years and over were twice likely to be probably demented compared with those aged 50–70 years (PR, 95% CI 2.42; 1.76–3.31). 71% of participants who were probably demented were females. Male participants were 1% less likely to be demented compared with their female counterparts (PR, 95% CI 0.99; 0.75–1.33). Table 3 shows bivariate logistic regression analysis of factors associated with probable dementia.

### Symptoms of probable dementia

In the initial analysis of symptoms score, the most common symptoms of probable dementia in the order of their severity were memory symptoms, (β co-efficient β 0.08, *p* < 0.001), physical symptoms (β 0.08, *p* < 0.001), sleep disturbances (β 0.81, *p* < 0.001) and emotions (β 0.04, *p* < 0.027). Table 2 shows symptoms of probable dementia.

**Table 2:** Symptoms of probable dementia.

Variables	Beta	Standard error	*p* value	Exp(B)	95% Confidence interval	
					Lower	Upper
Memory	0.08	0.01	**< 0.001**	1.08	1.06	1.11
Concentration	− 0.12	0.02	0.525	0.99	0.95	1.03
Physical symptoms	0.08	0.01	**< 0.001**	1.09	1.07	1.11
Emotions	0.04	0.02	**0.027**	1.04	1.00	1.08
Sleep disturbance	0.81	0.01	**< 0.001**	1.08	1.05	1.11
Others	− 0.001	0.03	0.984	1.00	0.94	1.07

Significant values are in bold.

### Factors associated with probable dementia

Bivariate analysis showed that probable dementia was significantly associated with age > 85 years (Prevalence ratio, PR = 2.42, *p* < 0.001), being married (PR = 0.70, *p* < 0.026), secondary/tertiary level of education (PR = 0.54, *p* < 0.004), occasional/non-believer (PR = 1.85, *p* < 0.001), smoker/ex-smoker (PR = 1.46, *p* < 0.013), occasional/no exercise (PR = 3.28, *p* < 0.001) and hypertension (PR = 0.70, *p* < 0.019). Probable dementia was not associated with sex, place of residence, formal employment, income level or use of alcohol. The final degree of association as determined by adjusted PR in the multivariable model, revealed that only older age (aPR = 1.88, *p* < 0.001) and occasional/non-believer (aPR = 1.61, *p* = 0.001) remained significantly related to probable dementia. Tables 3 and 4 show the bivariate and multivariable logistic regression analysis of factors associated with probable dementia respectively.

**Table 3:** Bivariate analysis of social demographic and patient characteristics associated with probable dementia (N = 264).

Variables	Normal (n = 142)	Probable dementia (n = 122)	PR (95% CI) 0.46(0.40–0.52)	*p*-value
	(f, %)	(f, %)		
Social demographic characteristics				
Age				
50–70 years	77 (54.2)	40 (32.8)	1.00	
71–85 years	61 (43.0)	63 (51.6)	1.49 (1.09–2.02)	0.011
> 85 years	4 (2.8)	19 (15.6)	2.42 (1.76–3.31)	**< 0.001**
Sex				
Female	100 (70.4)	86 (70.5)	1.00	
Male	42 (29.6)	36 (29.5)	0.99 (0.75–1.33)	0.99
Residence				
Rural	111 (78.2)	108 (88.5)	1.00	
Urban	31 (21.8)	14 (11.5)	0.63 (0.40–1.00)	0.048
Marital status				
Widowed	65 (45.8)	67 (54.9)	1.00	
Married	65 (45.8)	36 (29.5)	0.70 (0.51–0.96)	**0.026**
Single/divorced/separated	12 (8.5)	19 (15.6)	1.21 (0.87–1.67)	0.258
Level of education				
No formal/Primary education	98 (69.0)	105 (86.1)	1.00	
Secondary/Alevel/college/university	44 (31.0)	17 (13.9)	0.54 (0.35–0.82)	**0.004**
Formal employment				
Unemployed/retired	132 (93.0)	117 (95.9)	1.00	
Employed	10 (7.0)	5 (4.1)	0.71 (0.34–1.47)	0.356
Income level				
Low: Less than 500,000 shillings	136 (94.4)	118 (96.7)	1.00	
Good: Above 500,000 shillings	8 (5.6)	4 (3.3)	0.71 (0.32–1.60)	0.412
Practicing faith				
Practicing believer	103 (72.5)	54 (44.3)	1.00	
Occasional/Non-believer	39 (27.5)	68 (55.8)	1.85 (1.42–2.40)	**< 0.001**
Current living arrangement				
With family/friends/others	131 (92.3)	110 (90.2)	1.00	
Alone	11 (7.7)	12 (9.8)	1.14 (0.75–1.73)	0.528
History of smoking				
Non-smoker	130 (91.6)	101 (82.8)	1.00	
Smoker/Ex-smoker	12 (8.4)	21 (17.2)	1.46 (1.08–1.96)	**0.013**
Exercise				
Daily	59 (41.6)	19 (15.6)	1.00	
Occassionally	73 (51.4)	63 (51.6)	1.90 (1.23–2.93)	**0.004**
Not at all	10 (7.0)	40 (32.8)	3.28 (2.17–4.98)	**< 0.001**
Past medical history				
Hypertension				
No	78 (54.9)	85 (69.7)	1.00	
Yes	64 (45.1)	37 (30.3)	0.70 (0.52–0.94)	**0.019**
Alcoholism				
No	120 (84.5)	99 (81.2)	1.00	
Yes	22 (15.5)	23 (18.8)	1.13 (0.82–1.56)	0.454

PR is prevalence ratio aPR is adjusted prevalence ratio

Significant values are in bold.

**Table 4:** Multivariable analysis of socio demographic and patient characteristics associated with probable dementia.

Variable	aPR	95% CI	*p*-value
Age			
50–70 years	1.00		
71–85 years	1.31	(0.96–1.79)	0.089
> 85 years	1.88	(1.34–2.63)	**< 0.001**
Practicing faith			
Practicing believer	1.00		
Occasional/non-believer	1.61	(1.21–2.13)	**0.001**

Significant values are in bold.

### Knowledge of dementia

The study also found that eight percent of the participants had optimal knowledge of dementia.

Less than 40% of participants agreed that low level of education does not contribute to dementia; alcohol consumption is associated with dementia; when people with dementia repeat the same question or story several times, it is helpful to remind them that they are repeating themselves; and most people with dementia remember recent events better than things that happened in the past Table 5 describes the knowledge of dementia.

**Table 5:** Knowledge of dementia.

Variable	Frequency	Percent
	(N = 264)	(%)
Old age is strong predictor for dementia	210	79.6
It has been scientifically proven that lack of mental exercise can predispose a person to getting dementia	200	75.8
Low level of education does not contribute to dementia	65	24.6
Negative thinking can contribute to development of dementia	183	69.3
People with diabetes mellitus cannot develop dementia	191	72.4
Alcohol consumption is associated with dementia	85	32.2
If a person with dementia becomes alert and agitated at night, a good strategy is to try to make sure that the person gets plenty of physical activity during the day	217	82.2
People whose dementia is not yet severe can benefit from psychotherapy for depression and anxiety	223	84.5
If trouble with memory and confused thinking appears suddenly, it is likely due to dementia	171	64.8
Poor nutrition can make the symptoms of dementia	176	66.7
People in their 30 s can have dementia	201	76.1
A person with dementia becomes increasingly likely to fall down as the disease gets worse	125	47.4
When people with dementia repeat the same question or story several times, it is helpful to remind them that they are repeating themselves	102	38.6
Once people have dementia, they are no longer capable of making informed decisions about their own care	171	64.8
Eventually, a person with dementia will need 24-h supervision	162	61.4
Having high cholesterol may increase a person’s risk of developing dementia	206	78.0
Tremor or shaking of the hands or arms is a common symptom in people with dementia	169	64.0
Symptoms of severe depression can be mistaken for symptoms dementia	178	67.4
Trouble handling money or paying bills is a common early symptom of dementia	180	68.2
One symptom that can occur with dementia is believing that other people are stealing one’s things	239	90.5
When a person has dementia, using reminder notes is a crutch that can contribute to decline	84	31.8
Prescription drugs that prevent dementia are available	185	70.1
Having high blood pressure may increase a person’s risk of developing dementia	233	88.3
Genes can only partially account for the development of dementia	144	54.6
It is safe for people with dementia to drive, as long as they have a companion in the car at all times	215	81.4
Dementia cannot be cured	191	72.4
Most people with dementia remember recent events better than things that happened in the past	91	34.5
Overall level of knowledge		
Good	21	8.0
Moderate	215	81.4
Poor	28	10.6

## Discussion

### Sociodemographic characteristics

This study was conducted to determine the prevalence and factors associated with probable dementia and to describe the knowledge of the disease among older adults attending ROTOM health center-Mukono, Central Uganda. The mean age of the sample was 72.2 (range 50–101) years. This is similar to studies from Malaysia, Nigeria, Tanzania and Southwestern Uganda which reported mean age of 67, 74, 70 and 72 years respectively. Majority of the participants were females. Females were 71%, a proportion slightly higher than findings from these countries^4,7,14,15^. Research indicates that women live longer than men due to the protective role of oestrogen in women against the risks of cardiovascular disease, stroke and ageing. Oestrogen is shown to lower the low density lipoprotein (bad cholesterol), increase high density lipoprotein (good cholesterol) and is also an antioxidant^16^. The results of this study showed that over 80% of participants had lower level of education, rural residence, were formally unemployed and earned income less than 500,000 Ugandan Shillings (130 United States Dollars) per month. This is not surprising as the level of poverty in Uganda is high. Reports from Uganda Bureau of Statistics (UBOS 2019/2020) showed that 25% of persons in employment had completed either secondary or postsecondary training and the monthly median cash earnings for an employee in Uganda was 200,000 Ugandan Shillings (52 United States Dollars).

### Prevalence of probable dementia

The prevalence of probable dementia reported in this study is similar to that found in Malaysia^17^. The study reported that probable dementia was 46% in the sample. This study used the same tool, EDQ which was used and validated in a Malaysian study. EDQ is found not to be affected by patient’s educational background and cultural differences, and this explains the similarity of the findings in the two studies. However, the prevalence of probable dementia in this study is higher than that reported by earlier studies conducted in sub-Saharan Africa. Studies from Nigeria, Central African Republic, and Tanzania reported dementia prevalence of 25%, 10.1% and 6.4% respectively^18^, while a population-based study from Southwestern Uganda reported that a fifth of participants screened positive for dementia^7^. The observed differences may be attributed to differences in methods. This study used the EDQ which does not distinguish between patients with early dementia and mild cognitive impairment which may present with the same symptoms. Like any other screening tool, EDQ increases false positive rates.

### Symptoms of probable dementia

Dementia is diagnosed from its clinical symptoms. In the order of their severity, this study found that probable dementia was associated with presence of memory symptoms, physical symptoms, sleep disturbances and emotions. This confirms the findings from a Malaysian study which showed that the strongest predictor of possible early dementia was complaints of memory problems, followed by complaints of concentration, emotional problems and sleep disturbances^15^. Again, use of the same tool, EDQ which comprises of 20 clinical symptoms of dementia could possibly explain the similarity in the two studies. Similarly, studies from England and Brazil reported that memory loss and mood changes were the most commonly reported first symptoms of dementia^19,20^. This is also confirmed by a study from United States which stated that emotional/behavioural symptoms such as mood disturbances, apathy and agitation are a common accompaniments of dementia^21^. A community perceptions study in Southwestern Uganda stated that the persons with dementia commonly presented with forgetfulness, defecating and urinating on themselves, wandering away from home, going out naked and picking up garbage, consistent with findings in South Africa, Tanzania, Nigeria and Central African Republic^22^.

### Factors associated with probable dementia

The strongest predictor for dementia is older age. As expected, there was a significant association between older age and probable dementia. Participants who are older than 85 years were twice likely to get probable dementia compared to those who are 50–70 years (PR, 95% CI 2.42; 1.76–3.31; *p* < 0.001). This study confirms the findings by Mushi, Bradford, Owakuhaisa and de Jager, among others that advancing age is a strong non-modifiable predictor of dementia^3,4,22,23^. Annual life expectancy for both male and female in Uganda in 2022 is 64.06 years and has been increasing by 0.51% for the last 3 years. Life expectancy is expected to raise the prevalence in coming years if it keeps increasing. The risk of dementia due to increased age is complex. It is subject to various contributing factors across the life-course that may differ between those living in high and low-income regions^24^. There was a strong association between practicing faith and probable dementia. Participants who were occasional/non-believers were 85% more likely to develop probable dementia than those who were practicing believers (PR, 95% CI 1.85; 1.42–2.40; *p* < 0.001). This finding is consistent with the study by Henderson that infrequent religious service attendance was related to poorer cognitive functioning^25^ and Hill believes that religious practices such as singing, praying, attending sermons, studying scripture, and socialising with others during faith-based activities can maintain dense neocortical brain synapses and delay cognitive deterioration in the elderly^26^. ROTOM provides fellowship services for seniors. However, these spiritual activities are not person-centered and their impact in strengthening cognitive functioning is less felt among the elderly. Several studies in Africa also associated dementia with witchcraft, curses and other satanic causes. A study by Mushi, Rongai in rural Tanzania showed that participants believed their mental problems (including dementia) were caused by either ageing, chronic diseases, life stresses or witchcraft, and they stated Christian based healers and traditional healers as their healing pathway of choice^6^. Similarly, a study by Kakongi in rural Southwestern Uganda reported religious places of worship were the most common points of continuity of care for persons with dementia^27^.

### Knowledge of dementia

The study found a low level of knowledge of dementia among older adults in primary care. Eight percent of the participants had optimal knowledge of dementia. This confirms findings from several studies conducted in SSA. In South Africa, only 10% of persons with dementia reported knowing what dementia was^10^. Similarly, in Tanzania, dementia knowledge was low and symptoms are simply accepted as a problem of old age or supernatural causes^4^. There is a significantly poor knowledge of dementia among Undergraduate University Students in Uganda^11^.

### Limitations

The study was conducted in an institution providing services to the elderly. Therefore, findings may not be generalized at community/population level. The participants were sampled consecutively, therefore, there was lack of randomization and representativeness of the sample. There was a possibility of recall bias given that some participants might be experiencing memory loss and this might have also affected the knowledge scores. In addition, since some of the participants tested were illiterate elderly, there were higher chances of social desirability bias. Our results were from a screening test. However, a clinical diagnosis of dementia would have been ideal for comparison. We assumed that the Malaysian version of the EDQ is not affected by patient’s cultural and educational background and therefore was not validated in a Ugandan setting.

## Conclusions and recommendations

There is high burden of probable dementia among older adults attending ROTOM health center-Mukono. Probable dementia was 46.2% in a sample of older adults. The factors associated with probable dementia among older adults attending ROTOM health center-Mukono are older age and being an occasional/non-believer. There is low knowledge of dementia among older adults attending ROTOM health center-Mukono. Eight percent of participants had optimal knowledge of dementia. There is need to promote integrated early dementia screening, care and awareness program in ROTOM health center-Mukono.

Guidelines should be developed and/or adapted for screening, care, awareness and training on dementia in ROTOM health center-Mukono. Training on basic dementia care should be provided to healthcare and social workforce in ROTOM health center-Mukono. Person-centered religious support services should be provided to older adults attending ROTOM health center-Mukono. Further research should be conducted on development of national dementia strategy for early dementia screening, awareness and training in primary care facilities of Uganda. Further studies should be conducted to explore factors associated with knowledge of dementia among the general population.

## Data Availability

The datasets used and/or analyzed during the current study are available from the corresponding author on reasonable request.

## Abbreviations

DKAS: Dementia Knowledge Assessment Scale
EDQ: Early Dementia Questionnaire
GDS: Geriatric Depression Scale
MGH: Mukono General Hospital
PR: Prevalence ratio
PHC: Primary Health Care
ROTOM: Reach One Touch One Ministries
SSA: Sub-Saharan Africa
UBOS: Uganda Bureau of Statistics
WHO: World Health Organization
